# A Scoping Review of Exercise Oncology Trials to Inform Best Practice Recommendations for Exercise in Older Adults (65+ years) Living with and Beyond Cancer

**DOI:** 10.1016/j.jgo.2025.102334

**Published:** 2025-08-09

**Authors:** Kerri Winters-Stone, Mary Crisafio, Christopher Chalmers, Gabrielle Meyers, Elizabeth Eckstrom, Kristin L. Campbell

**Affiliations:** 1 Division of Oncological Sciences, Knight Cancer Institute, School of Medicine, Oregon Health & Science University, Portland OR; 2 Division of Hematology/Oncology, Knight Cancer Institute, School of Medicine, Oregon Health & Science University; 3 Division of General Internal Medicine & Geriatrics, Oregon Health & Science University, Portland, OR; 4 Department of Physical Therapy, Faculty of Medicine, University of British Columbia, Vancouver, Canada

**Keywords:** aging, physical activity, older adult, cancer survivor

## Abstract

**Introduction:**

We conducted a scoping review of published controlled exercise oncology trials to inform the development of best practice guidelines for delivering exercise to older cancer survivors.

**Materials and Methods:**

Eligible articles reported on controlled trials that allocated people ≥65 years old at enrollment and a history of cancer to ≥ 1 arm of structured exercise training lasting ≥ 4 weeks that was aimed to improve a health-related outcome. We extracted and summarized data on trial design, basic characteristics, trial outcomes, eligibility criteria, pre-exercise medical clearance, intervention characteristics, feasibility (accrual, retention, adherence, compliance), safety and tolerance.

**Results:**

Out of 1790 articles identified, 1784 were excluded, yielding six eligible studies. Three trials tested a combined aerobic + resistance training program, one trial tested aerobic exercise only, one trial included resistance training only, and one trial compared an arm of resistance training to an arm of aerobic training. Main health-related outcomes were aerobic capacity (n=1) and physical function (n=5). Four out of six studies required medical clearance prior to participation and several studies excluded physical (n=3) and/or cognitive limitations (n=3). Trial accrual ranged from 21%–37%, adherence ranged from 48%–84%, and retention ranged from 61%–77%. Compliance with exercise training was rarely tracked or reported. One serious adverse event (syncope leading to hospitalization) possibly attributed to exercise was reported across all six trials.

**Discussion:**

There are very few studies available to inform evidence-based exercise guidelines for older adult cancer survivors. In turn, consensus-based guidelines could provide recommendations so that older cancer survivors receive appropriate exercise guidance.

## Introduction

By 2040, 24% of all cancer survivors will be ages 65–74, 31% will be ages 75–84, and 18% will be 85 and older, but our healthcare system may not be prepared to meet the needs of this growing population of older survivors [1]. The combined effects of cancer treatment and aging can accelerate the development of comorbidities, disabilities, and fall-related injuries [1]. Cancer treatment can alter health and functioning in ways similar to aging by causing fatigue, weakness, cognitive decline, dependence, and disability while also causing unique treatment-related toxicities like chemotherapy-induced peripheral neuropathy (CIPN) or vestibular dysfunction [2], [3]. These effects are exacerbated by increasing levels of pain, depressive symptoms, and social isolation that is associated with cancer and cancer treatment, further diminishing quality of life [4], [5], [6]. Exercise could provide a low-cost, accessible, and efficacious strategy to improve functioning and quality of life for this growing population[1]. However, exercise programs must consider the unique abilities, needs, and preferences of older adults living with and beyond cancer (herein, referred to as older cancer survivors) and the combined complexities of aging and chronic illness.

Current exercise guidelines for cancer survivors from the American College of Sports Medicine (ACSM) and the American Society of Clinical Oncology (ASCO) recommend modest levels of exercise (e.g., 30 minutes of moderate-intensity aerobic or resistance exercise three times per week) to improve cancer-related health outcomes, including physical functioning and health-related quality of life [7], [8]. However, a noted limitation of both recommendations was the inability to issue age-specific guidelines for older cancer survivors due to the lack of clinical trial evidence specific to this population. The National Comprehensive Cancer Network also provides exercise guidelines for all cancer survivors, with a few additional recommendations that are relevant to older survivors, such as engaging in balance exercise for lowering fall risk [9], [10]. However, clinical practice remains largely bereft of specific guidance to include exercise in care plans for older cancer survivors, which may contribute to their low engagement in recommended levels of exercise [11].

While the evidence base matures, development of consensus-based best practice recommendations could fill the practice gap that impedes the implementation of exercise in standard of care for older cancer survivors. The ACES (Advancing Capacity to Integrate Exercise into the Care of Older Cancer Survivors) initiative was funded from a National Cancer Institute-funded effort to build infrastructure in cancer and aging (1R21CA280996) and has an aim to develop consensus-based exercise recommendations for older cancer survivors. The goal of the recommendations is to complement the existing ACSM guidelines on exercise for cancer survivors by adding specificity for older (65+ years) survivors on pre-exercise evaluation and assessment, and exercise prescription, programming, tolerance, and safety. In developing recommendations, ACES followed an evidence-to-decision making framework that included extracting information from the extant literature [12]. For this purpose, we conducted a scoping review of published controlled exercise oncology trials that were designed for cancer survivors over the age of 65. Our objectives were to describe the consideration of pre-exercise evaluation, participant inclusion/exclusion criteria, exercise prescription, programming and delivery, outcome selection, and safety and tolerance in controlled exercise trials to help inform best practice recommendations.

## Methods

This scoping review adhered to the Preferred Reporting Items for Systematic Reviews and Meta-Analysis (PRISMA) guidelines. The PRISMA checklist for scoping reviews is available in Supplementary File 1.

### Eligibility

Randomized controlled trials with one or more arms of planned aerobic, resistance, and/or movement exercise lasting at least four weeks in duration that enrolled individuals over the age of 65 and with a history of cancer were eligible for this review. Trials had to report on at least one physiologic or patient-reported outcome, such as physical or cognitive functioning. We excluded trials that involved alternative or complimentary forms of movement since the FITT prescription cannot be applied to these modalities (i.e., some forms of yoga, mind-body movement), trials that focused on physiotherapy, therapeutic or rehabilitative approaches to address a specific impairment, and trials that focused on behavior change or physical activity behavior only.

### Search Strategy and Trial Selection

Trials published between January 2000 and December 2024 were identified from electronic databases: OVID MEDLINE. The search strategy was developed in consultation with a health sciences librarian and is included in Supplementary File 2. All searches were conducted between September through December 2024. No authors were contacted for additional information or sources.

**Figure 1:**
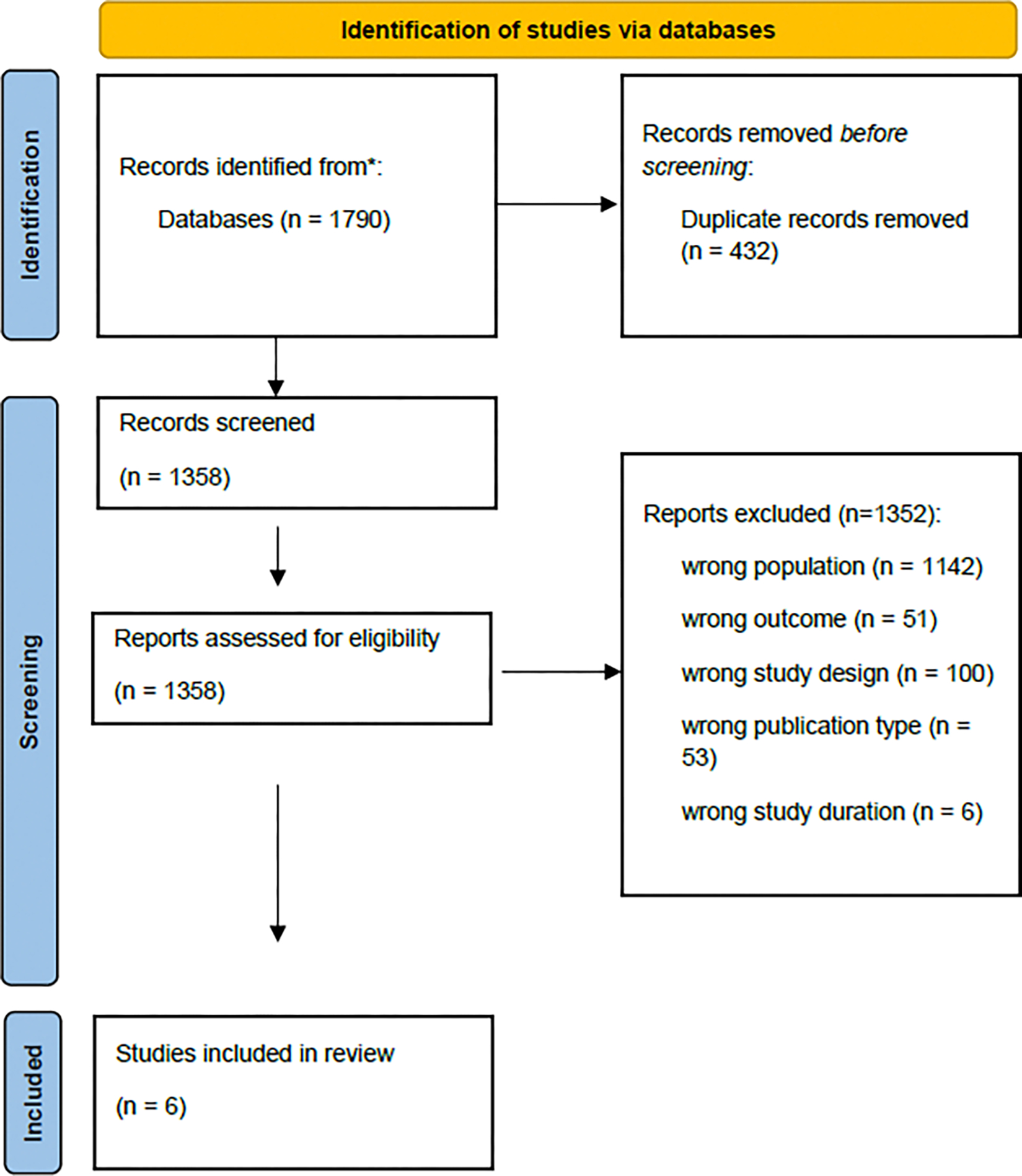
Identification and Selection of Studies for Scoping Review Source: Page MJ, et al. BMJ 2021;372:n71. doi: 10.1136/bmj.n71.

Citation details (i.e., authors, title, journal name, year of publication, volume, issue number, page numbers, and trial abstract) were uploaded to the Rayyan Systematic Review software [13]. A single author (MC) reviewed the title list and removed all duplicates. Two authors (MC & CC) independently screened the title and abstracts for inclusion criteria. The full texts of remaining articles were independently reviewed against inclusion and exclusion criteria (MC & CC). Disagreements were resolved through discussion with a third author (KWS). The process of article selection, including reasons for exclusion, is illustrated in Fig 1.

### Data Extraction

Data extraction was completed by two authors (MC & CC). We specifically extracted information about: trial sample size, age, cancer type(s), treatment modalities, intervention timing relative to treatment, intervention mode, control condition, health-related outcomes, eligibility criteria, pre-exercise medical evaluation, instructor characteristics, intervention details and procedures (frequency, intensity, time, type, and progression [FITT(P)]), trial accrual, adherence, compliance tracking methods, trial retention, intervention modifications, and adverse event reporting.

## Results

### Trial Selection

The database search yielded 1790 records. After duplicates were removed (n=850) and inclusion criteria were assessed (n=940), six full-text articles were included (Fig 1). One article by Olsen et al. reported on a sub trial from a larger randomized controlled trial of geriatric interventions in frail, older (≥70 years) adults with colorectal cancer, where participants found to be physically frail at baseline were referred to an exercise program [14]. It was included as we felt that the sample represented a clinical population recruited for a trial specifically designed for older cancer survivors and that the trial design and outcomes could provide relevant information that aligned with other controlled studies.

### Trial Design

All trials allocated participants to at least one arm of structured exercise training (Table 1). Four trials had one intervention arm [15], [16], [17], [18]. Two trials had two intervention arms where one compared similar training programs delivered as structured exercise to a game-based version of the similar training versus standard care [19]. The other trial compared aerobic to resistance training against a flexibility control condition [20].

### Trial Population and Eligibility Criteria

Per eligibility criteria, all trials enrolled participants who were at least 65 years of age at enrollment (Table 1), with mean ages ranging from 68.8 ± 3.4 to 77.5 ± 6.7. Three trials targeted single cancer types – prostate (n=1) [19], breast (n=1) [20], and colorectal (n=1) [18] – while the remaining three studies enrolled survivors of mixed cancer types [15], [16], [17]. Sample sizes ranged from 14–114 participants with a mean sample size of 40. One trial intervened on patients prior to urologic surgery [15], four intervened during treatment (chemotherapy or hormone therapy) [16], [17], [18], [19] and one intervened posttreatment [20].

Trial inclusion and exclusion criteria were extracted and summarized (Table 2), including age and cancer diagnosis, requirements for medical clearance before participation, pre-enrollment physical and cognitive functioning levels, and medical contraindications. Two trials limited their inclusion to patients with an Eastern Cooperative Oncology Group performance status (ECOG PS) of ≤2 [17], [18]. Three trials cited cognitive limitations as exclusion criterion, determined either by objective assessment (n=1) or by self-report (n=2) [17], [20]. Trials excluded for various contraindications to exercise including uncontrolled hypertension (n=1), [16] use of beta-blockers (n=1) [16], unstable medical disease (n=1) [17], exercise orthopedic limitations (n=1)[16], brain metastases (n=1), or unstable bone metastases (n=1) [17]. Four out of six trials required medical clearance for exercise participation as part of their eligibility criteria [15], [16], [17], [20]. Only one trial had eligibility criterion to target low functioning participants who were deemed physically frail [18].

### Intervention Characteristics

The trial exercise interventions were delivered by an exercise physiologist (n=2) [19], [20], physiotherapists (n=2) [17], [18] or physician (n=1) [15]; one trial did not report who delivered the intervention [16]. Two trials reported that professional leading the exercise intervention had to have experience delivering exercise to patients with cancer [18], [20] and one of these trials also required experience delivering exercise to older adults [20], while the other four trials did not specify experience [15], [16], [17], [19]. Three trials delivered the intervention in both group plus individual sessions [17], [18], [20], with one having an additional six months of individual exercise at home after supervised training stopped [20]. For group exercise, the size of group was reported in one trial and was 12 per class [18]. The remaining three trial interventions were conducted individually [15], [16], [19]. Only one trial was fully unsupervised [19], with the remaining trials being either fully supervised (n=1) [15] or a combination of supervised + unsupervised sessions (n=4) [16], [17], [18], [20]. Four trials were conducted in a university exercise lab [15], [16], [18], one at an academic health science university [20], one in a cancer center at a medical facility [17], and one was completely home-based [19].

Exercise intervention details are described according to the frequency, intensity, time, and type (FITT) prescription, with the added detail of exercise progression (P) (Table 3). Intervention duration ranged from 4–72 weeks, with a median duration of 12 weeks. Three trials tested a combined aerobic + resistance training program [16], [17], [20], one trial tested aerobic exercise only [15], one trial included resistance training only [18], and one trial compared an arm of resistance training to an arm of aerobic training [19]. One trial compared aerobic + resistance training to similar training plus a balance component [19]. Frequency of weekly exercise sessions (including all modalities) ranged from 3–5+ sessions each week, with most trials prescribing three weekly sessions.

Aerobic exercise intensity and its measurement varied across the five trials [15], [16], [17], [19], [20]. Three trials used heart rate reserve (HRR), [16], [19], [20] with intensities across trials ranging 35% to 75% HRR, and one trial used maximal load (Watts) [15] ranging from 100% to 115% of their maximal load determined at baseline. Resistance exercise intensity was prescribed as a percentage of body weight [16] or repetition maximum (RM) or % of body weight in a weight vest [20]. Aerobic exercise time varied across trials. One trial [15] used five one-minute bouts of intense exercise, while others [16], [20] ranged from 20 to 60 minutes per session. Two trials [17], [19] either did not report exercise duration or individualized it. Resistance training time was typically reported using a set and repetition scheme. Aerobic exercise was completed with cycle ergometer [15], walking (with or without treadmill)[16], [17], [19], or low-impact aerobic movements using the upper and lower body (marching, side steps, knee lifts, etc.) [20]. Resistance exercise was completed with free weights (dumbbells, barbells, weight vests, etc.) [16], [20], resistance bands [18], [19], [20], machines [17], [18], body weight[18], or a % of body weight in a weight vest [20]. Exercise intervention progression was described in all six trials, but the level of detail provided was variable and, in some cases, might not be replicable.

### Primary Trial Outcomes

For studies that identified a primary outcome (n=4), it was either aerobic capacity (n=1) or physical function (n=3) (Table 1). All but one study [15] included an objective measure of physical function, which included one or more of the following: Short Physical Performance Battery (n=2), chair stand test (n=5), and/or stair climb test (n=1) (Table 1). No study included an objective or patient-reported measure of cognitive function. A full list of trial outcomes and their measures can be found in Table 1.

### Accrual, Adherence, Compliance, Retention, and Modifications

Two trials reported on accrual rate reported, which ranged from 21% to 37% [17], [20], while four trials did not report accrual data (Table 4.) [15], [16], [18], [19]. Adherence to prescribed exercise sessions was reported in five trials and ranged from 48% to 84%, with an average adherence rate of 79% [15], [17], [18], [19], [20]. All trials utilized standardized methods for prescribing exercise intensity, described as % of HRR, RPE, and pedometers; however, it is unclear if these methods were utilized to measure exercise compliance. One trial monitored and reported compliance to aerobic and resistance exercise by heart rate reserve and rating of perceived exertion scales, respectively [20]. Retention was reported in four trials and ranged from 61% to 77% [17], [18], [19], [20]. Two trials described how an exercise program would be modified based on participant tolerance, but did not provide enough detail to be useful for implementation in practice [16], [20]. Marechal et al. reported modifications related to adapting the resistance program to avoid any tension around the port-a-cath region, while Winters-Stone et al. reported modifications for individuals who could not fully comply with the prescribed program and were provided alternative exercises within each modality.

### Safety and Tolerance

Methods for reporting safety and adverse events (AEs) were often vague or poorly described, with only two trials indicating that this information was obtained verbally by a physiotherapist [18] at the onset of the AE or by the exercise trainer who verbally queried for AEs each week [20]. Adverse events definitely or possibly attributable to exercise were reported in five trials, and across these studies there were a total of seven mild, three moderate, and one serious AE (Table 4.). The single serious AE (fainting during exercise intervention warm up, later diagnosed with cardiotoxicity) was reported in one trial [18] but was never clearly attributed to the exercise intervention. One trial did not report monitoring for AEs [16].

## Discussion

The intention of our scoping review was to summarize the evidence base of controlled exercise oncology trials in older cancer survivors to help inform the development of exercise guidelines for this underserved population. Specifically, the goal of the ACES initiative was to develop recommendations that complement existing ACSM guidelines on pre-exercise evaluation and assessment, and exercise prescription, programming, safety, and tolerance in older cancer survivors. There remains an extremely limited number of studies to draw from and limited information in published studies to inform this effort and thus, many gaps remain for developing evidence-based recommendations. However, the available evidence could be considered along with other sources of information (e.g., professional expertise, other exercise guidelines) to drive consensus-based guidelines and to inform the design and reporting of future research to strengthen the evidence base moving forward.

Determining whether medical evaluation and/or clearance should be sought prior to the initiation of exercise training is a crucial decision that weighs the risks of an adverse event from exercise against a potential barrier of requiring medical clearance that might deter older cancer survivors from starting an exercise program. Older cancer survivors may be at higher risk of adverse events from exercise due to age and comorbidities but may also benefit tremendously from regular exercise. Among the six trials reviewed, four required clearance from a medical provider prior to exercise and two of these had additional exclusion criteria for physical and/or cognitive limitations [15], [16], [17], [20]. Two studies excluded participants for standard exercise contraindications like uncontrolled hypertension [16] or unstable disease [17], while two trials excluded participants with low physical functioning by ECOG status [17], [18] and three trials for low cognitive functioning[17], [19], [20]. A primary goal of pre-exercise medical clearance is to reduce the risk of a serious exercise-related adverse event. In the five trials that reported AEs, only one reported a single serious AE possibly attributed to exercise training in a medically fragile population already screened for functional limitations. The incidence of moderate AEs, when reported, was very low, whether or not medical clearance was obtained, consistent with trials in cancer survivors of any age [7]. It is possible that requiring medical clearance or excluding for other medical conditions or physical or cognitive limitations precluded enrollment of more medically complex patients who may have higher exercise risks. The current ACSM exercise guidelines for all cancer survivors recommend that the ACSM pre-participation guidelines for evaluating the need for medical clearance for non-cancer comorbidities should be applied in cancer survivors along with the National Comprehensive Cancer Network recommendations for specific cancer-related health conditions [9], [10]. Whether or not older cancer survivors have additional health considerations that warrant medical clearance for exercise cannot be determined by the current evidence base reviewed here. Adding additional conditions that would require medical clearance prior to starting an exercise program should be weighed against the potential barrier that this may pose to engaging older cancer survivors in exercise that may be safe if appropriately prescribed and delivered.

Current ACSM exercise guidelines recommend 30-minutes or more of aerobic and/or resistance exercise two to three times per week to improve several cancer-related health outcomes, including perceived physical functioning [7]. In these guidelines, there was insufficient data to inform evidence-based recommendations for outcomes that may be particularly salient for older cancer survivors, including falls, cognitive functioning, and mobility [21]. Other exercise guidelines for the general population of older adults, including ACSM, American Family Physician, and the National Comprehensive Cancer Network exercise guidelines for all cancer survivors, state that balance exercises should be performed by older adults or cancer survivors deemed at risk of falling but issue no specific prescription [7], [9], [10]. These same guidelines recommend that older adults and/or cancer survivors engage in twice weekly flexibility exercises. Further, the International Conference on Frailty and Sarcopenia Research (IFSCR) [22] and National Strength and Conditioning Association (NSCA) [23] guidelines for exercise in older adults emphasize the need to target major muscle groups involved in function and mobility and including activities that directly simulate daily activities, such as a sit-stand or step-up exercise can directly transfer to an improvement in activities of daily living (ADLs).

Of the interventions tested in the six trials reviewed, the primary modality was aerobic and/or resistance training. All reported improvements in aerobic capacity or physical functioning, consistent with the broader ACSM guidelines. No trial specifically tested balance or flexibility training, except for two trials that used stretching as a placebo control condition [16], [20]. Two trials integrated a limited number of balance exercises into primary resistance + aerobic training programs but did not measure changes in specific balance outcomes or falls [17], [19]. Trials testing aerobic interventions alone or with resistance exercise used cardio machines which may not mimic functional movements, while interventions using outdoor walking or aerobic dance programs may include more functional movements[17], [18], [19], [20]. Half of the resistance trials used machines which may build strength but not in functional movement patterns, whereas the other half used free weight exercise with bands, dumbbells, and/or weight vests and two of these included functional movements[16], [19], [20]. One trial specifically employed functional movements for the resistance training exercises and reported significant improvements in physical functioning compared to aerobic or placebo stretching arms. The single trial to focus on frail older survivors reported significant improvements in physical functioning from a resistance training program that used both machine-based exercises to improve strength and functional exercises using resistance bands [18].

Among the current literature in older cancer survivors, there is a lack of attention to the design of exercise interventions that aim to reduce falls, mobility disability and dependence, and/or that follow the exercise recommendations for older adults. This may limit the development of specific exercise prescriptions for older cancer survivors that could have a measurable impact on reducing disability and injuries from falls. Future studies should consider designing trials to improve other primary endpoints that are practically and clinically meaningful to older adults including cognitive functioning, depression, loneliness, and quality of life.

Additional programming considerations for exercise interventions that have implications for best practice recommendations include the setting, trainer qualifications, and degree of supervision including trainer to participant ratios for group-based programs to ensure safe and effective delivery of exercise. Interventions were mostly delivered at an academic facility under supervision, but most also included additional unsupervised sessions done at home. Only one trial had participants exercise on their own at home throughout the intervention period [19]. Time, transportation, cost, and access can be barriers to exercise, particularly for older cancer survivors who may have financial, mobility, and access constraints. Given the low adverse event rate across studies, it is possible that under the right conditions unsupervised exercise can be safely performed by older cancer survivors. The increase in the use of live, remote, supervised exercise training via videoconferencing could allow for a more accessible way to deliver supervised individual or group exercise that could be considered in future trials in older cancer survivors [24], [25], [26], [27].

Interventions were mostly overseen by persons with an exercise or medical certification, with only two studies stating that they required interventionists to have experience delivering exercise to cancer survivors [18], [20] and only one also requiring experience in older adults[20]. While these trainer characteristics are highly desirable to optimize delivery of exercise, the workforce with these skill sets remains limited [28], [29] and access to skilled professionals may create another barrier to getting older cancer survivors to engage in recommended levels of exercise [18]. Best practice recommendations regarding the degree of supervision, setting, and trainer preparation for exercise delivery in older cancer survivors will have to balance the tradeoffs between the perceived risks and the potential barriers to exercise training.

The feasibility metrics of controlled trials can provide insight for implementation in clinical and community practice but also point to research gaps that can be filled in future studies. Accrual rates were rarely reported but ranged from 21% to 37%, suggesting that trials may not enroll a truly representative sample of older cancer survivors due to exclusion for physical and/or cognitive limitations and/or due to barriers to participation in a facility-based clinical exercise trial, such as time and transportation. Limiting exclusion criteria or even focusing on a more limited population, such as Olsen et al.[18], may ensure that trial outcomes can generalize to the broader older cancer survivor population, who may be sicker or frailer than typical trial populations. Reducing barriers to participation in clinical trials such as time and travel is now more possible with decentralized clinical trials where supervised exercise could be delivered remotely.

Adherence rates to exercise sessions averaged 79% and retention in trials averaged 70%, though a few trials were much lower. The same barriers to enrollment may affect adherence and retention rates. Future studies should report either in published protocols, supplementary files, or separate reports the tactics used to optimize accrual, adherence, retention, and success rates to inform best practice recommendations for engaging older cancer survivors in sustained exercise training. Measuring compliance to intensity of training provides information about whether older cancer survivors can achieve prescribed levels of training and can be a safety technique to watch for poor tolerance or over-exertion. One trial in older women cancer survivors reported using RPE and a heart rate monitor to assess compliance to target training zones for supervised aerobic and resistance training throughout the intervention period[20]. While it is possible that other trials monitored exercise compliance, methods were not reported and thus it is unclear if the samples reached target training intensities. Improving use and reporting of compliance metrics in future trials will better confirm the levels of exercise that are safe and tolerable for older cancer survivors and inform best practice exercise recommendations.

One of the biggest concerns for exercise expressed by older cancer survivors is perceived tolerance and safety [30], [31], [32]. Older survivors may be wary that overexertion might cause an adverse event or worsening symptoms, such as pain. Providers may also be less inclined to recommend exercise to their older patients out of safety concerns. Adverse event reporting from clinical trials provides data to support the risks and safety of exercise and must be consistently reported in clinical exercise trials. Among six studies in this review, only one failed to report AEs[16] and there was only one serious AE related to the intervention, in an on-treatment, frail, advanced cancer population where an adverse outcome might be more expected [18]. Consistent and systematic reporting of AEs in future clinical exercise studies is imperative to establish the tolerance and safety of exercise for older cancer survivors. Routine monitoring and unbiased systematic recording and reporting of AEs by grade and attribution can provide rigorous data to establish safety of exercise [33]. Likewise, AEs can be used to monitor tolerance over a trial and quantify whether and how dose reductions or other modifications improved tolerance. AEs can also be tracked in community and clinical practice. With these data, the evidence bases for both safe and effective exercise can be used to better reassure older cancer survivors and providers that exercise can be safe when delivered appropriately.

Our scoping review was intended to provide evidence that could inform exercise guidelines specific to older cancer survivors that would fill gaps in current national recommendations. Unfortunately, there remains a limited number of controlled trials designed for older adults to draw from. Among reviewed trials, some required upfront medical clearance which may or may not be necessary to ensure safety and may present a potential barrier for implementing exercise in practice. Several trials excluded physical or cognitive limitations, which are more common in older adults with cancer, in turn limiting generalizability of findings. Trials did not test exercise modalities that are recommended for older adults, including balance, flexibility, and functionally based movements, yet older cancer survivors have a greater risk of falls and disability that may be better addressed by adding specificity to training programs. It is likely that trials did not include fully representative samples by excluding participants with limitations or barriers to participation, but adherence and retention in most studies was moderately good. Most importantly, the exercise programs tested were safe, addressing a major reason survivors may hesitate to engage in exercise and providers may hesitate to recommend exercise to their older patients. Clearly, more studies that are specifically designed for older cancer survivors are needed. Trials need to consider inclusive eligibility criteria and ways to reduce barriers to participation, like fully decentralized trials, to engage a representative sample. Interventions should be specifically designed to address the most pressing physical and cognitive limitations that older survivors face. Detailed reporting and rigorous assessment of compliance, tolerance, and safety through published protocols and trial outcome reports will also provide data that can inform best practice recommendations and foster implementation in clinic and community settings. While the current evidence will be useful in forming consensus-based recommendations, it is imperative that exercise oncology trials focus on this underrepresented population of cancer survivors because evidence-based guidelines that address the needs of older survivors will have the greatest impact on the ability of clinical and community practice to serve the largest proportion of people living with and beyond cancer.

## Supplementary Material

1

2

## Figures and Tables

**Table 1.** Basic Study Design, Sample, Intervention Features, and Outcomes in Reviewed Studies

Citation	Sample Size	Age (years): Median [IQR] or {Range}, or Mean (±SD)	Cancer Type(s)	Intervention Timing Relative to T reatment	Intervention Mode(s)	Control Condition	Health-Related Outcome(s)
Blackwell et al. (2020)	40	72 (4)	Prostate, Bladder, Kidney	Before (pre-surgical)	Aerobic	Standard Care	-MCID in anaerobic threshold (VO_2AT_): CPET* -Blood Pressure -Body Composition: DXA -Muscle Architecture: DXA
Maréchal et al. (2019)	14	68.8 (3.4)	Breast and Colon	During	Aerobic + Resistance	Flexibility	-Aerobic Fitness: 6MWT -Upper Body Endurance/Strength: arm curl test & hand grip strength -Physical Function: 30s-CST -Leg Strength: 1-RM sitting squat
Mikkelsen et al. (2022)	84	IG: 72.1 [67.3–74.5] CG: 71.5 [68.5–75.3]	Pancreatic, Biliary Tract, and Non-Small Cell Lung	During	Aerobic + Resistance	Standard Care	-Physical Function: 30s-CST* -Aerobic Fitness: 6MWT -Upper Body Strength: hand grip -Body Composition: DXA & BIA -QOL: EORTC QLQ-C30 -Clinical Outcomes: treatment toxicities & hospital admissions
Olsen et al. (2023)	40	HAG: 77 {71–84} LAG: 75 {70–83}	Colorectal	During	Resistance	N/A*	-BMI -Physical Function: ECOG PS & ADL -Physical Function: 30s-CST, SCT -Leg Strength: 1-RM Leg press -Upper Body Strength: 30s-ACT -QOL: EORTC QLQ-C30 & QOL ELD14
Sajid et al. (2016)	19	Aerobic + Resistance: 77.5 (6.7) Aerobic + Resistance + Balance: 75.7 (9.5) Control: 71.8 (5)	Prostate	During	Aerobic + Resistance vs. Aerobic + Resistance + Balance	Standard Care	-Physical Function: SPPB Score* -Aerobic Capacity: 6MWT -Muscular Strength: hand grip & 1-RM chest press -Body Composition: DXA
Winters-Stone et al. (2022)	114	70.9 (5.1)	Breast	After	Aerobic vs. Resistance	Flexibility	-Physical Function: SPPB, SF-36 physical function subscale, & LLDFI* -Aerobic Fitness: 6MWT -Upper Body Strength: 1-RM chest press -Lower Body Strength: 1-RM leg press -Flexibility: back scratch & chair sit- and-reach

Age described in years as median [interquartile range; IQR] or {range}, or mean (± standard deviation); Intervention group (IG); Control group (CG); High adherence group (HAG); Low adherence group (LAG), (*) Indicates the study’s primary outcome(s) and measure(s), (MCID) minimal clinically important difference, (Vo_2at_) oxygen consumption at anaerobic threshold, (CPET) cardiopulmonary exercise test, (DXA) dual-energy X-ray absorptiometry, (6MWT) 6-minute walk test, (30s-CST) 30 second chair-stand-test, (1-RM) 1 repetition maximum, (PASE) Physical Activity Scale for the Elderly, (BIA) Bioelectrical impedance, (EORTC QLQ-C30) European Organization for Research and Treatment of Cancer Quality of Life Questionnaire Core 30, (BMI) body mass index, (ECOG PS) Eastern Cooperative Oncology Group performance status, (SCT) stair climb test, (30s-ACT) 30-second arm curl test, (SPPB) short physical performance battery, (SF-36) 36-Item Short Form Health Survey, (LLFDI) Late-Life Function and Disability Instrument, (CHAMPS) Community Healthy Activities Model Program for Seniors

**Table 2.** Eligibility Criteria for Study Samples in Reviewed Studies

Citation	≥65 Years Old	Cancer Diagnosis	Medical Clearance	Treatment Type	Exclusion Criteria			
					Contraindications to Exercise	Physical Function	Cognitive Function	Other
Blackwell (2020)	✔	✔	✔	-Open prostatectomy--Radical Cystectomy— La pa rosco pic nephrectomy -Robotically assisted radical prostatectomy	No criteria	No lower limit	No criteria	Unable to adhere to study protocol (travel, etc.)
Marechal (2019)	✔	✔	✔	Receiving a systemic treatment that started 12 weeks or less prior to baseline	Exercise orthopedics limitations, uncontrolled hypertension, use of beta-blockers	No lower limit	No criteria	None
Mikkelsen (2022)	✔	✔	✔	Had received or were scheduled to receive first-line palliative systemic treatment (chemotherapy, immunotherapy, or targeted therapy)	Not having any uncontrolled and/or extensive brain metastases, unstable medical disease, or bone metastases that pose a risk of pathological fractures	ECOG PS score ≥3	Not having any physical, mental, or cognitive conditions that hindered participation based on safety	
Olsen (2023)	✔	✔	No criteria	Stage II to IV CRC receiving adjuvant or first-line palliative/downstaging chemotherapy	No criteria	ECOG PS score ≥3 Frailty according to the G8 questionnaire (14/17)	No criteria	coexisting cancer within five years
Sajid (2016)	✔	✔	No criteria	Receiving ADT for at least 3 months	No criteria	No significant physical impairment (i.e. unable to walk 4 m as part of SPPB measured walk)	<5 errors on the short portable mental status questionnaire	
Winters-Stone (2022)	✔	✔	✔	≥2 years post-chemotherapy and/or radiation therapy	No criteria	No lower limit	No cognitive difficulties that would preclude answering the survey questions, participating in the performance tests, or giving informed consent	

(ECOG PS) Eastern Cooperative Oncology Group performance status, (SPPB) short physical performance battery, (ADT) androgen deprivation therapy

**Table 3.** Summary of Intervention Characteristics in Reviewed Studies

Citation	Intervention Modality	Functional Movements and/or Balance Exercises	Duration of Intervention	Characteristics of Person Delivering Exercise			Group of Individual	Degree of Supervision	Exercise Setting(s)	Exercise Protocol (FITT(P))
				Qualification	Cancer Experience	Older Adult Experience				
Blackwell (2020)	Aerobic	NR	4 weeks	Medically qualified doctor	NR	NR	Individual	Supervised	University Exercise Lab	**F:** 3–4× weekly **I:** 100–115% of the maximal load (watts (W)) ^†^ **T:** 5× 1-minute intervals **T:** Cycle ergometer **(P):** *An increase in wattage was implemented at the mid-way point of training to maintain exercise intensity with progression*
Marechal (2019)	Aerobic + Resistance	NR	12 weeks	NR	NR	NR	Individual	Supervised & Unsupervised	University Exercise Lab & Home	**F:** 3× weekly. 2/3 sessions in-lab; 1/3 sessions in-lab or at home. **I:** Aerobic-70–75% of HRR; Resistance-50–65% of perceived 1- RM **T:** Aerobic- 40 minutes; Resistance- 2–3 sets, 10–15 reps **T:** Aerobic-treadmill; ResistanceMajor muscle groups were trained with and without dumbbells. **(P):** Aerobic- Participant’s exercise intensity and time was ramped for the first 4 weeks of the intervention to the described intensity and time; Resistance- NR
Mikkelsen (2022)	Aerobic + Resistance	Balance: Exercises performance as part of warm up (walking with changing directions, walking on toes and heels, or balance board)	12 weeks	Physiotherapist	NR	NR	Group & Individual	Supervised & Unsupervised	Cancer Center & Home	**F:** Aerobic-1× weekly; Resistance- 2× weekly **I:** Aerobic-Individualized; Resistance-15-RM (S1-S2), 12-RM (S3-S13), 10-RM (S14-S24) **T:** Aerobic-Individualized step count; Resistance-60 minutes **T:** Aerobic-Walking; Resistance- Chest press, abdominal crunch, leg press, leg curl, leg extension, lower back extension, and low row. **(P):** Aerobic- Individualized goal setting and evaluation. Resistance-15-RM (S1-S2), 12-RM (S3-S13), 10-RM (S14-S24). Number of sets: 2 (S1-S6), 3 (S7-S24)
Olsen (2023)	Resistance	Functional: Chair to stand, bridging, & back extension	12 weeks	Physiotherapist	Yes	NR	Group & Individual	Supervised & Unsupervised (optional)	University Exercise Lab & Home	**F:** In-Lab-2× weekly; Home-1× weekly **I:** In-Lab-15-RM (S1-S2), 12-RM (S3-S13), 10-RM (S14-S24); Home- 3×10-RM **T:** In-Lab-60 minutes, 2×15 (S1-S2), 2×12 (S3-S6), 3×12 (S7-S13), 3×10 (S14-S24); Home-30 minutes **T:** In-Lab-Leg press, chest press, knee extension/flexion, low row, sit ups, back extension, arm curl, pull down, & chest press (machines and resistance bands were used); Home-Chair to stand, bridging, sit ups, back extension, arm curl, pull down, & chest press (resistance bands) **(P):** In-Lab-Set and repetition progression scheme: 2×15 (S1-S2), 2×12 (S3-S6), 3×12 (S7-S13), 3×10 (S14-S24); Home: NR
Sajid (2016)	Aerobic + Resistance	Balance: Wii-Fit technology	6 weeks	Exercise physiologist	NR	NR	Individual	Unsupervised	Home	**F:** Aerobic- ≥5× weekly; Resistance- ≥5× weekly **I:** Aerobic- 60%-70% HRR & 3–5 RPE; Resistance- 3–5 RPE. Participants received 3 colored bands increasing in tensile strength. Set and rep scheme was designated on an individual basis **T:** Aerobic- NR, variable to step count; Resistance- **T:** Aerobic- Walking; Resistance- Banded exercises including: bicep curl, tricep extension, overhead press, rows, chest press, internal and external rotation, lateral and front raises, horizontal adduction, and abduction **(P):** Aerobic- 5% to 20% increase of step count each week, aiming for 10,000 steps per day; Resistance- Instructed to progressively increase from low to moderate bands, and increase from their baseline sets and repetitions to a maximum of 4 sets of 15 repetitions for each exercise daily
	Aerobic + Resistance + Balance	NR	6 weeks		NR	NR	Individual	Unsupervised	Home	**F:** Balance-NR **I:** Balance-NR **T:** Balance-NR **T:** Balance-NR **(P):** Balance-NR This exercise program was individually tailored and designed to deliver a similar mode, intensity, and duration of exercise as EXCAP [Aerobic and Resistance study arm] with the addition of a balance component.
Winters-Stone (2022)	Aerobic	NR	72 weeks	Exercise physiologist or certified fitness instructors	Yes	Yes	Group & Individual	Supervised (First 12-months) & Unsupervised (Last 6-months)	University Exercise Lab & Home	**F:** 3× weekly **I:** 35–65% HRR **T:** 20–45 minutes for aerobic, 60 minutes total **T:** Low-impact dance aerobic exercises including marching, side steps, knee lifts, upper arm and dance movements. **(P):** Over 9 months the duration of aerobic exercise progressed from 20 to 45 minutes of active training and intensity progressed from 35% to 65% of estimated heart rate reserve (HRR)
	Resistance	Functional: lower body- chair stands, front, back, & lateral lunges, and calf raises, upper body- push/pull and raises	72 weeks		Yes	Yes	Group & Individual	Supervised (First 12-months) & Unsupervised (Last 6-months)	University Exercise Lab & Home	**F:** 3× weekly **I:** Upper body: 2–3 sets of 10–15- RM of each exercise. Lower body exercises were performed with a weighted vest that increased from 1% of pt body weight up to 10%. **T:** 2–3 sets of 10–15-RM, 60 minutes total **T:** 5 upper body and 5 lower body exercises including chair stands, front, lateral, & backward lunges, calf raises, one-arm row, chest press, front & lateral shoulder raise, and push up. **(P):** Upper body- weight was progressively increased to maintain a 10–15-RM. Lower body: weighted vest that was gradually increased from 1% of a participant’s body weight up to 10%, or as tolerated.

(†)100–115% of the maximal load (watts (W)) reached during their initial cardiopulmonary exercise test, (NR) not reported, (HRR) heart rate reserve, (S#-S#) describes session # through session #, (#-RM) # of repetition(s) maximum, (RPE) rating of perceived exertion, (wk) week, Frequency, intensity, time, type, & progression (FITT(P)).

**Table 4.** Study accrual and adherence, intervention compliance, retention, modifications, and safety in reviewed studies

Citation	Accrual	Adherence	Compliance	Retention	Modifications	Exercise Related Adverse Events (AEs)
Blackwell (2020)	NR	84%	NR	NR	NR	Two mild adverse events of leg pain were reported after an exercise session, attributed to the exercise intervention. Both cases were self-limiting and required no intervention.
Marechal (2019)	NR	NR	NR	NR	Adaptions to the resistance program were made to avoid any tension in the region of the Port-A-Cath	NR
Mikkelsen (2022)	37%	Aerobic: 75% Resistance: 69%	NR	75%	NR	-Five mild adverse events of bruises and feeling sick/dizzy after exercise were reported, all attributable to exercise intervention -Three moderate adverse events of: -Swollen knee after exercise was reported, attributable to exercise intervention -Back pain in days following exercise, diagnosed with osteoporotic spinal compression, possibly attributable to exercise intervention -Pain & instability in knee after initiating exercise intervention, diagnosed with spinal stenosis, possibly attributable to exercise intervention
Olsen (2023)	NR	48%	NR	73%	NR	-One serious adverse event of fainting during warm up portion of exercise session, later hospitalized for cardiotoxicity, authors do not report if attributable to exercise intervention
Sajid (2016)	NR	61%	NR	61%	NR	No adverse events occurred
Winters-Stone (2022)	21%	72%	RPE + Heart Rate Monitor	77%	Individuals who could not fully comply with the prescribed program due to pre-existing orthopedic limitations that restricted movement were provided alternative exercises within each modality.	No serious adverse events occurred in either study arm. Mild & moderate adverse events were not reported

(NR) not reported, (RPE) rating of perceived exertion, (1-RM) one repetition maximum

## Data Availability

The datasets generated during and/or analyzed during the current study are available from the corresponding author on reasonable request.
