# Supplementary material for: A Scoping Review of Exercise Oncology Trials to Inform Best Practice Recommendations for Exercise in Older Adults (65+ years) Living with and Beyond Cancer
Source: J Geriatr Oncol. Author manuscript; Available in PMC 2026 Aug 9. (PMC12413888; doi:10.1016/j.jgo.2025.102334)
Supplement: Supplementary file 1 [file NIHMS2104186-supplement-1.docx]

**Supplementary File**

**Database:** Ovid MEDLINE(R) ALL <2000 to December 23, 2024> Search Strategy:

**Search String:** 1 exp Neoplasms/ (3906749) 2 exp cancer survivors/ (9242) 3 exp antineoplastic protocols/ (161964) 4 exp antineoplastic agents/ (1257779) 5 1 or 2 or 3 or 4 (4560519) 6 exp EXERCISE/ (251096) 7 exp Physical Fitness/ (36708) 8 exp Exercise Therapy/ (64459) 9 exp EXERCISE MOVEMENT TECHNIQUES/ (10496) 10 exp sports/ (219350) 11 6 or 7 or 8 or 9 or 10 (400840) 12 5 and 11 (13330) 13 exp Musculoskeletal Physiological Phenomena/ (1165281) 14 exp musculoskeletal system/ (1598333) 15 13 or 14 (2417485) 16 5 and 15 (179273) 17 12 and 16 (10866) 18 ((cancer* or neoplas* or oncolog* or carcino* or adenocarcino* or tumor* or tumour* or malig* or metasta* or leukem* or leukaem* or lymphom* or melanom* or sarcom*) adj15 ((physical* or muscle* or musculo*) adj3 (fit* or strength* or stamin* or endur* or capac*) adj10 (exercis* adj3 (aerobic* or therap* or treat* or capac* or tolera* or capab*)))).mp. [mp=title, book title, abstract, original title, name of substance word, subject heading word, floating sub-heading word, keyword heading word, organism supplementary concept word, protocol supplementary concept word, rare disease supplementary concept word, unique identifier, synonyms, population supplementary concept word, anatomy supplementary concept word] (99) 19 17 or 18 (10897) 20 limit 19 to english language (10486) 21 limit 19 to abstracts (9618) 22 20 or 21 (10758) 23 limit 22 to "all aged (65 and over)" (3301) 24 limit 22 to "middle age (45 to 64 years)" (4233) 25 24 not 23 (1412) 26 limit 23 to (systematic reviews pre 2019 or systematic reviews) (119) 27 limit 25 to (systematic reviews pre 2019 or systematic reviews) (40) 28 limit 23 to (controlled clinical trial or randomized controlled trial) (732) 29 limit 25 to (controlled clinical trial or randomized controlled trial) (359) 30 random*.mp. (1720557) 31 limit 24 to clinical trial, all (1177) 32 limit 25 to clinical trial, all (414) 33 30 and 31 (1012) 34 30 and 32 (359) 35 28 or 33 (1124) 36 29 or 34 (374) 37 35 not 26 (1118) 38 36 not 27 (373)
